# Da Vinci robot-assisted laparoscopic retroperitoneal debridement for lumbar septic spondylodiscitis: A two-case report

**DOI:** 10.3389/fsurg.2022.930536

**Published:** 2022-09-07

**Authors:** Jichao Ye, Hao Liu, Xumin Hu, Jinteng Li, Liangbin Gao, Yong Tang

**Affiliations:** ^1^ Department of Orthopedics, Sun Yat-sen Memorial Hospital of Sun Yat-sen University, Guangzhou, China; ^2^ Department of Urology, Sun Yat-Sen Memorial Hospital of Sun Yat-sen University, Guangzhou, China; ^3^ Department of Orthopedics, The Eighth Affiliated Hospital of Sun Yat-sen University, Shenzhen, China

**Keywords:** robotics, laparoscopy, pyogenic spondylodiscitis, lumbar spine, Da Vinci surgical system®

## Abstract

The anterior approach is one of the widely used surgical treatments for lumbar spondylodiscitis, but it has the disadvantages of large trauma and a high incidence of complications. Our experiences suggested that the laparoscopic retroperitoneal approach could be effective to overcome those disadvantages of the anterior approach. Herein, we report two cases of successfully treated lumbar pyogenic spondylodiscitis using a robot-assisted laparoscopic retroperitoneal approach. The technique utilizes a robot that allows a laparoscopic retroperitoneal approach while offering excellent high-definition images of three-dimensional vision. After the operation, both patients achieved good formation and fusion of the vertebrae. Preliminary evidence suggests that the robot-assisted laparoscopic retroperitoneal approach may be feasible for the treatment of lumbar spondylodiscitis.

## Introduction

Pyogenic spondylodiscitis refers to the infection of intervertebral discs, cartilage endplates, and adjacent vertebrae (1). Surgical treatments of lumbar spondylodiscitis mainly include anterior and posterior approaches (1). The advantages of the anterior approach include debridement under direct vision, ensuring the removal of necrotic tissue, and effectively protecting the anterior lumbar vascular as well as other important structures, while preservation of posterior column integrity is conducive to stability after spinal surgery (2). However, trauma and high incidence of complications are two obvious disadvantages of the anterior approach (2–4). Our previous experience suggests that retroperitoneal endoscopy can effectively reduce the trauma of the anterior approach and improve the operative effect (5). Moreover, robots may be ideal surgical assistants in spinal surgery as they can achieve superior levels of precision. Multiple studies have shown that the robot-assisted technique is more accurate than the conventional method in spine surgery (6, 7). Based on the cognition above, we performed two robot-assisted laparoscopic retroperitoneal procedures for the treatment of lumbar pyogenic spondylodiscitis, which are reported as follows.

## Case reports

This study was approved by the institutional review board at the authors’ institution. Written informed consent was obtained from each subject. Further, these subjects and/or their families were informed that data from the cases would be submitted for publication, after which they gave their consent. This study was conducted in accordance with the principles of the Declaration of Helsinki and with the laws and regulations of China.

### Case 1

The patient, a 78-year-old man, was admitted to the hospital because of lumbar pain. Two months before admission, the patient had suffered from lumbar pain without any precipitating cause, and it became obvious during nighttime, aggravated after activity. There was no fever, lower limb–radiating pain, or other symptoms. Symptomatic treatment in the local hospital did not improve the symptoms. Physical examination revealed lower lumbar spinous process tenderness and percussion pain. Lumbar spine flexion, extension, and lateral flexion were limited. There was no abnormal muscle strength and muscle tension in both lower extremities. The straight leg raising test was negative, as also the Babinski sign. The patient had a history of diabetes for 5 years and was treated with oral hypoglycemic drugs.

**Figure 1:**
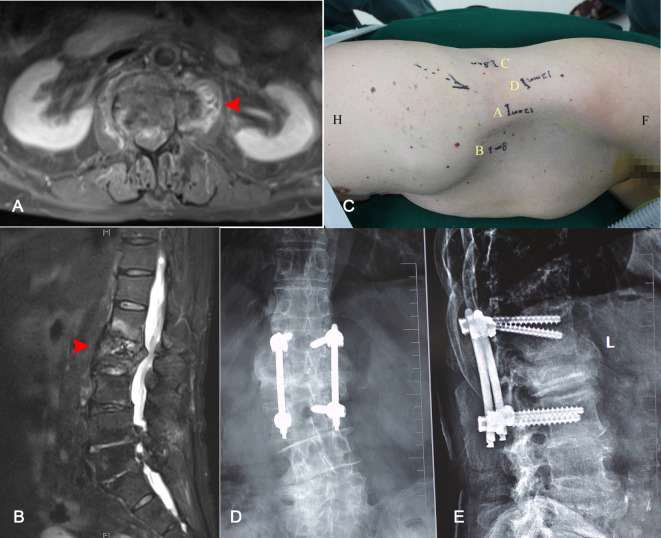
Cross section (**A**) and sagittal (**B**) T2-weighted MRI demonstrated destruction at the L1/2 intervertebral space and a partial L2 vertebral body (red arrow). The location of the working channel was planned before operation (**C**). Thirty months after the operation, the x-ray of the lumbar spine performed in the positive position (**D**) and the lateral position (**E**) showed the formation and fusion of the vertebrae.

A routine blood test showed WBC 12.61 × 10^9^/L, ESR 16 mm/h, and CRP 7.75 mg/L. The T-SPOT test was negative. No abnormalities were found upon the tumor series examination. Lumbar spine x-ray showed L1/2 intervertebral disc destruction; lumbar CT three-dimensional reconstruction showed lesions in the L1/2 vertebral body, intervertebral disc, and surrounding soft tissue lesions, the results from lumbar MRI scan for L1/2 vertebral body, intervertebral disc, and surrounding soft tissue were considered to show infectious lesions (Figures 1A,B).

**Figure 2:**
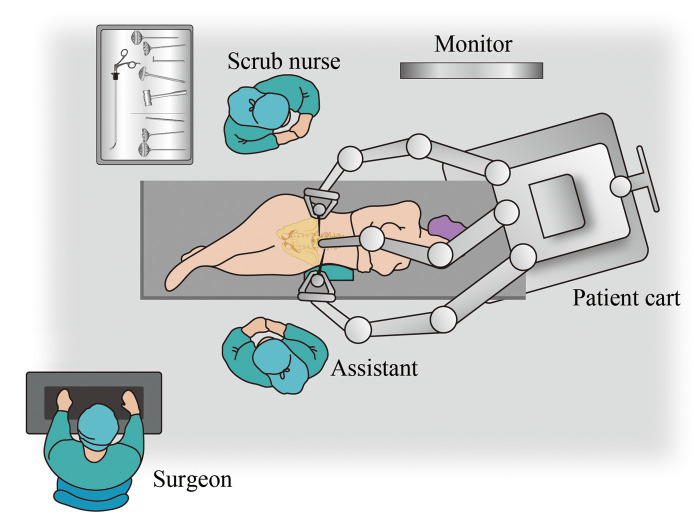
Robotic instrumentation, personnel, and operating room setup for the laparoscopic retroperitoneal approach.

**Figure 3:**
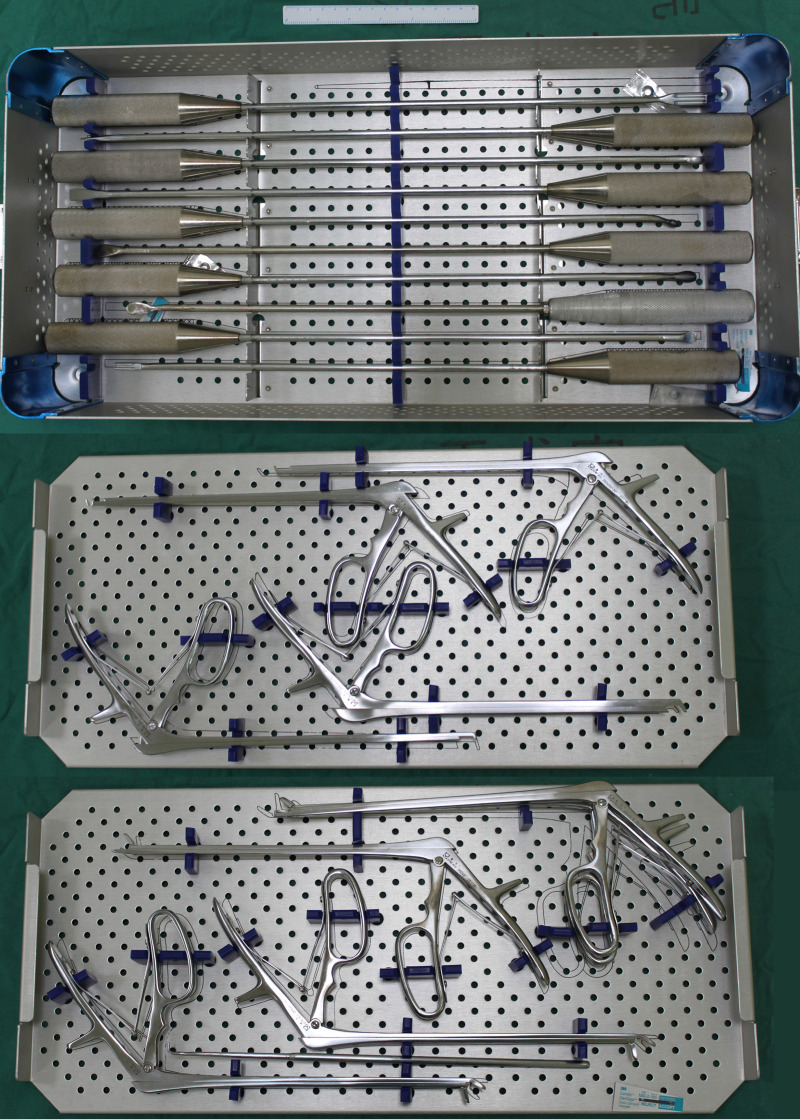
Operative instruments for robot-assisted laparoscopic retroperitoneal debridement surgery. Scale bar = 15 cm.

A robot-assisted laparoscopic retroperitoneal approach procedure was performed. General anesthesia was performed by using tracheal intubation, ambulatory blood pressure was monitored by using arterial intubation, and dynamic CO_2_ partial pressure was monitored. The patient lay on the right lateral decubitus using the Trendelenburg position with low head and low foot. The Da Vinci XI Surgical System (Intuitive Surgical, Sunnyvale, CA, USA) was placed on the head side of the patient, with the medial axis aligned to the retroperitoneal space (Figure 2). The placement of the working channel was planned before operation (Figure 1C). After routine disinfection and towel laying, the upper two transverse fingers of the iliac spine in the middle axillary line were taken as the A point to create the lens arm channel. The skin was cut about 1.5 cm longitudinally, the muscular layer and lumbar fascia were separated bluntly, and the retroperitoneal space was separated bluntly by using the fingers. A self-made air sac was inserted and injected with 600 ml of air. Then, 1 cross finger under 11 ribs and 8 cm away from point A was taken as the robotic arm channel (B point), and an 8 mm trocar for the robot was placed. The posterior line of the armpit 8 cm from the A point was taken as the C point, which was the second robotic arm channel. Point D as the auxiliary hole was between A point and C point, and a 12 mm trocar was placed. Another 12 mm trocar was placed at the A point, and CO_2_ gas was added to establish the retroperitoneal air chamber after suturing the skin. The lens arm was connected to the trocar at the A point, and the two mechanical arms were connected to the trocar at the B and C points, respectively. After fixing the lens and the lens arm properly, the Maryland forceps and unipolar bending shears were fixed with the two robotic arms, respectively, and the instruments were moved into the operation area under direct vision. The peritoneum was pushed bluntly to the abdomen and the space behind the retroperitoneum was enlarged. To identify the psoas major muscle, unipolar scissors were used to separate between the psoas major fascia and peritoneum in order to expose important anterior structures of the vertebral body such as the ureter and aorta. A C-arm x-ray machine was used to guide endoscopic titanium clips to locate the diseased vertebrae, unipolar scissors were used at the anterior edge of the psoas major muscle to separate the muscle tissue and retracted psoas major muscle to expose the diseased vertebrae. Next, the paravertebral pus was cleared, the L1/2 intervertebral disc fibrous ring was cut, the necrotic nucleus pulposus and bone tissue were cleared, and local irrigation was repeated. The drainage tube was placed at the lesion through the auxiliary cannula, and the robotic arm was pulled out. L1 and L3 vertebral bodies were fixed by using a percutaneous pedicle screw system under the guidance of the C-arm. All the operative instruments are shown in Figure 3, which included the nucleus pulposus forceps kit, lamina rongeur kit, stripper series, curette series, endplate scraper series, and osteotome series. The length of the working section of the above-mentioned tools ranged from 25 to 35 cm, while the diameter ranged from 5 to 10 mm. These parameters ensured that the above-mentioned surgical instruments could pass smoothly through the 12 mm trocha.

Postoperative pathological examination showed suppurative inflammation, and pus and tissue culture were negative. The patient was treated with broad-spectrum antibiotics and vacuum drainage, and the drainage tube was removed 5 days after the operation. The patient was discharged 1 week after the operation, and his lumbar pain was relieved. After discharge, the thoracolumbar scaffold and oral antibiotics were recommended for 3 months (vancomycin 15 mg/kg IV q12 h for 4 weeks and levofloxacin 500 mg PO for 8 weeks). Thirty months after the operation, x-ray examination showed intervertebral bone formation and fusion (Figures 1D,E).

### Case 2

The patient, a 57-year-old woman, complained of low back pain for 20 days, and the pain was obvious during the night. Body temperature fluctuated between 37.5°C and 38.6°C.

**Figure 4:**
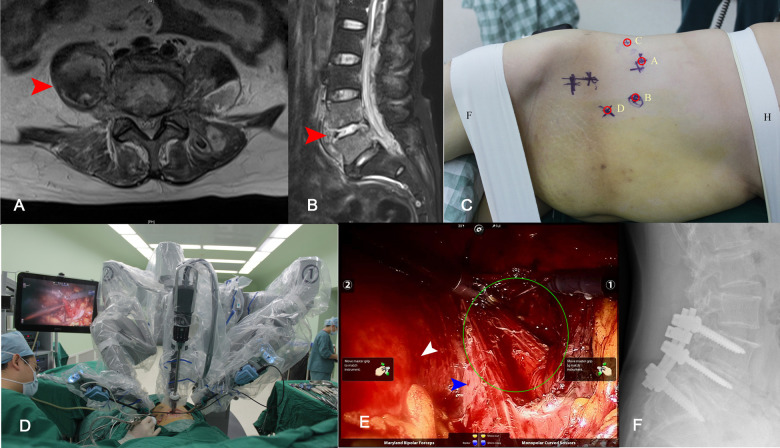
Cross section (**A**) and sagittal (**B**) T2-weighted MRI demonstrated destruction at the L4/5 intervertebral space, and a huge abscess formation at the paravertebral (red arrow). The location of the working channel was planned before the operation (**C**), the abscess and L4/5; the intervertebral disc tissue was removed during operation (**D, E**). Endoscopic view of the procedure, peritoneum (white arrow), psoas muscle (blue arrow), and abscess (green circle) (**E**). Twenty-eight months after operation, x-ray showed a good internal fixation position and fusion of L4/5 intervertebral (**F**).

A routine blood test showed WBC 11.03 × 109/L, ESR 53 mm/h, and CRP 45.9 mg/L. The T-SPOT test was negative. A lumbar spine MRI scan revealed infectious lesions in the L4/5 vertebral body, intervertebral disc, and surrounding soft tissues (Figures 4A,B). An operation was scheduled.

The robot was placed on the patient’s caudal side, and the mid-axis aligned to the retroperitoneal space. A 15-mm incision was made 6 cm above the iliac ridge in the anterior axillary line as the A point, the retroperitoneal space was split, and an endoscope was placed. The B point and the C point were made on both 8 cm sides of the A Point to serve as mechanical arm channels. The D-point was made at L4/5 for auxiliary tools (Figure 4C). Abscess and L4/5 disc tissue were removed intraoperatively (Figures 4D,E). One-staged posterior L4-S1 pedicle screw fixation was performed. The result of postoperative tissue bacterial culture indicated Staphylococcus aureus. The antibiotic regime after the operation was vancomycin for 4 weeks and levofloxacin for 8 weeks. Lumbar x-ray 28 months after surgery showed a good internal fixation position and fusion of the L4/5 intervertebral space (Figure 4F).

## Discussion

Laparoscopy technology is an important branch of minimally invasive surgery. However, its application in spinal surgery is progressing slowly. In 1991, Obenchain first used the transabdominal approach to perform anterior L5/S1 laparoscopic discectomy (8). McAfee reported retroperitoneal laparoscopic discectomy and interbody fusion in 1998 (9). In 1999, Olinger reported the retroperitoneal laparoscopic treatment of lumbar fractures that one-stage posterior pedicle screw fixation was performed, and anterior laparoscopic bone grafting and plate fixation were performed through a retroperitoneal approach (10). The lesions of these two cases were both located in the middle and anterior columns of the lumbar vertebra. This extraperitoneal approach facilitated the visualization and removal of infectious lesions as well as preventing the infection from spreading to the abdominal organs. Similarly, since 2009, laparoscopic surgery has been applied in the treatment of lumbar tuberculosis through an extraperitoneal approach. One-stage anterior debridement and bone grafting plus anterior/posterior internal fixation have achieved good results (11). Thus, laparoscopic retroperitoneal debridement is a rational strategy for treating lumbar septic spondylodiscitis located in the anterior vertebral body.

The robot system is based on laparoscopy surgery (12). It provides high-definition images of three-dimensional vision for the surgeon so that the surgeon can identify the essential anatomical structures such as the abdominal aorta, inferior vena cava, common iliac artery/vein, psoas muscle, lumbar sympathetic trunk, and superior hypogastric plexus (SHP). (13). This clear vision significantly helps surgeons to avoid injury above anatomical structures and reduce bleeding during operations. Moreover, the camera system is controlled by the robotic arm with a stable vision and a more flexible viewing angle. The level of freedom of the robotic arm and Endo-Wrist of the robot exceeds the limit of human hands, and it can perform precise movements continuously without fatigue and error during the psoas muscle separation procedure (14). In summary, the application of robots can help improve laparoscopy surgery.

In 2013, Lee et al. first reported surgery by a robot wherein the patient underwent intraperitoneal approach anterior L5/S1 discectomy plus bone grafting and internal fixation *via* laparoscopy (14). In the extraperitoneal approach, the extraperitoneal space is relatively narrow, which is not conducive to the deployment of the robotic arm, and hence there are few reports about the extraperitoneal approach of the robot (15). As mentioned above, laparoscopic retroperitoneal debridement is a rational strategy for treating lumbar septic spondylodiscitis located in the anterior vertebral body to avoid the risk of peritonitis, in contrast to the transperitoneal approach. The Da Vinci robot can further expand these advantages. Compared with conventional laparoscopy, the Da Vinci robot provides higher-resolution images of three-dimensional vision for the surgeon. This is particularly important for the surgeon to clearly identify the essential anatomical structures such as the abdominal aorta, inferior vena cava, common iliac artery/vein, psoas muscle, lumbar sympathetic trunk, and superior hypogastric plexus (SHP) during the operation, since lumbar septic spondylodiscitis may make the retroperitoneal space and organs edema and adhesion which may be difficult to be identified and separated sometimes. Higher-resolution images can significantly reduce bleeding and the incidence of organ injury during operations. Moreover, the flexibility and stability of the robotic arm can further reduce the possibility of injuring vital organs as well as removing infectious lesions more effectively. Finally, we recorded the following experiences after the operations were performed successfully: (1) The space of the retroperitoneal is small, and it is easy to penetrate the peritoneum when placing point B robotic arm Trocar. When the self-made balloon expands the retroperitoneal space, 600 ml of air is injected. After removing the balloon, the peritoneum is pushed forward as bluntly as possible with the index finger, and Trocar is placed under the guidance of the index finger. (2) Obstructed by the robotic arm, the position of the assistant hole is far away from the lesion, which puts the forward higher requirement for the tool for spine surgery by laparoscopy. (3) When the location of the lesion cannot be identified during the operation, the titanium clip can be temporarily placed, the robotic arm can be removed, and the C-arm x-ray machine can be used to guide the localization in order to reduce the separation and injury of soft tissue.

At present, although this surgical technique is very efficient for soft tissue, it has limited ability for bone and other hard tissues (16). Since there are still no matching instruments for the Da Vinci robot system to handle bony structures, we have to clear the necrotic nucleus pulposus and bone tissue manually. Thus, stability and flexibility cannot be qualified during the above operative process (16). In addition, the operation cost is expensive, and the surgeon needs special training. Thus, the application value of this surgical technique in spine surgery needs further research and discussion. Moreover, developing matching instruments for the Da Vinci robot system to handle bony structures is one of our future research orientations.

## Conclusion

As the number of lumbar anterior approach surgeries increased in recent years, especially in mainland China, robot-assisted surgery is still an inevitable development direction in this field. This paper shows that the lumbar operation *via* the retroperitoneal anterior approach is feasible, safe, and flexible. Given the development of manufacturing technology and the decrease in the cost related to this kind of operation in the near future, the author is optimistic about the application of robots in spine surgery.

## Data Availability

The raw data supporting the conclusions of this article will be made available by the authors, without undue reservation.
